# Stroke Secondary Prevention: Everyone’s Business

**DOI:** 10.3390/healthcare10112236

**Published:** 2022-11-08

**Authors:** Maggie Lawrence, Olive Lennon, James Faulkner

**Affiliations:** 1 Research Centre for Health (ReaCH), Glasgow Caledonian University (GCU), Glasgow G4 0BA, UK; 2 School of Public Health, Physiotherapy and Sports Science, University College Dublin, Dublin D04 V1W8, Ireland; 3 School of Sport, Health and Community, Faculty of Health and Wellbeing, University of Winchester, Winchester SO22 5NR, UK

Stroke secondary prevention is everyone’s business and requires cohesive working across the multiprofessional team and beyond. Consequently, a broader definition of stroke secondary prevention than that typically seen in research papers, clinical guidelines and policy documents is required.

In 2015, INSsPiRE, an international network of stroke secondary prevention researchers, was established to raise awareness of secondary prevention in the context of multiprofessional stroke rehabilitation and recovery. (https://www.gcu.ac.uk/aboutgcu/academicschools/hls/research/researchgroups/livingwithstroke/insspire (accessed on 1 November 2022)).

INSsPiRE’s first undertaking was to achieve expert consensus on a definition of stroke secondary prevention that applies across disciplines and extends beyond pharmacological and surgical interventions to also encompass what might be more broadly termed as ‘lifestyle’ issues:

Non-pharmacological and non-surgical stroke secondary prevention supports and improves long-term health and well-being in everyday life and reduces the risk of another stroke, by drawing from a spectrum of theoretically informed interventions and educational strategies. Interventions to self-manage modifiable lifestyle risk factors are contextualized and individualized to the capacities, needs, and personally meaningful priorities of individuals with stroke and their families

[1].

Arguably, the consensus definition is not easily accessible, but it does represent a first step in the work being undertaken to support the development of an extensive and credible evidence base. Additionally, in this *Healthcare* Special Issue, “Healthy Living and Risk Reduction after TIA and Stroke”, we showcase research that advances our understanding of—and the evidence-base for—non-pharmacological non-surgical stroke secondary prevention in this broad sense. The international collaborative efforts published here set the scene and highlight new directions for policy and practice in stroke secondary prevention across disciplines and across practice contexts.

Hall et al. (2022), in their scoping review of contemporary stroke guideline documents and clinical audits, shine a spotlight on the current lack of clear direction for clinicians in addressing secondary prevention beyond pharmacological or surgical management [2]. This work highlights the imperative to raise awareness amongst researchers and clinicians of the urgent need for high-quality holistic secondary prevention stroke care initiatives that will help improve outcomes (medical, psychological, social, and societal) for stroke patients and their families. Clinical guidelines and government policies are informed by the best available contemporaneous evidence. In the field of stroke secondary prevention, this contemporaneous evidence has been sparse and heterogenous to date, and likely to be considered as being of low quality, and is often reported along with advice to treat such evidence ‘with caution’.

The qualitative study by Lennon et al. (2022) published in this Special Issue reports from the stroke patients’ perspectives the acceptability and feasibility of holistic secondary prevention initiatives which address a broad range of lifestyle issues, whilst also highlighting unmet needs including the management of fatigue, and self-management of secondary preventive medication [3]. Wang et al. (2022), in reporting a mixed methods study, focus on mood disorders as a risk factor for stroke and recurrent stroke patients. Improving the management of such symptoms can not only help reduce the risk of stroke recurrence, but can also provide the motivation stroke patients need to be able to better engage with the self-management of risk factors, as well as rehabilitation and recovery more generally [4]. The paper by Patomella et al. (2021) explores the perspectives of people post-TIA regarding the acceptability and accessibility of digital technology in the context of self-management of risk reduction following TIA, which is increasingly relevant in a post-pandemic context [5]. With these papers, along with a further five high quality research papers that make up this Special Issue, we are furthering our understanding of healthy living and risk reduction regarding stroke as we showcase research at the forefront of this critically important topic.

Many health professionals working in the specialty of stroke may perceive themselves to be too busy with current workloads to address secondary prevention issues with patients and their families/significant others/caregivers, particularly where workloads do not extrinsically include secondary prevention (beyond medication prescription). The editorial team hope readers of this Special Issue will now look more critically at the risk factors for stroke recurrence and implement changes, however small, to their current practices. Looking beyond stroke to the wider field of public health, we can find examples of approaches to healthy lifestyle counselling that demonstrate that the implementation of secondary prevention interventions need not add to perceived role burden. Such initiatives are seen in the Public Health Scotland (2013) campaign Every Step Counts (https://youtu.be/AbcNRrmlMbc0 (accessed on 1 November 2022)) and in the Irish campaign Making Every Contact Count (https://www.hse.ie/eng/about/who/healthwellbeing/making-every-contact-count/ (accessed on 1 November 2022)). With a little practice and forethought, secondary prevention initiatives can be incorporated seamlessly into patient/family interactions by medics, AHPs, and nurses alike, and can help change awareness and practice by acknowledging that stroke secondary prevention is indeed everyone’s business.
